# Mathematical modeling predicts pathways to successful implementation of combination TRAIL-producing oncolytic virus and PAC-1 to treat granulosa cell tumors of the ovary

**DOI:** 10.1080/15384047.2023.2283926

**Published:** 2023-11-27

**Authors:** Justin Le Sauteur-Robitaille, Powel Crosley, Mary Hitt, Adrianne L. Jenner, Morgan Craig

**Affiliations:** a Department of Mathematics and Statistics, Université de Montréal, Quebec, Canada; b Sainte-Justine University Hospital Research Centre, Montréal, Québec, Canada; c Department of Oncology, University of Alberta, Edmonton, Canada; d School of Mathematics, Queensland University of Technology, Brisbane, Australia

**Keywords:** Granulosa cell tumors, ovarian cancer, oncolytic virus, mathematical modeling, treatment optimization

## Abstract

The development of new cancer therapies requires multiple rounds of validation from *in vitro* and *in vivo* experiments before they can be considered for clinical trials. Mathematical models assist in this preclinical phase by combining experimental data with human parameters to provide guidance about potential therapeutic regimens to bring forward into trials. However, granulosa cell tumors of the ovary lack a relevant mouse model, complexifying preclinical drug development for this rare tumor. To bridge this gap, we established a mathematical model as a framework to explore the potential of using a tumor necrosis factor-related apoptosis-inducing ligand (TRAIL)-producing oncolytic vaccinia virus in combination with the chemotherapeutic agent first procaspase activating compound (PAC-1). We have previously shown that TRAIL and PAC-1 act synergistically on granulosa tumor cells. In line with our previous results, our current model predicts that, although it is unable to stop the tumor from growing in its current form, combination oral PAC-1 with oncolytic virus (OV) provides the best result compared to monotherapies. Encouragingly, our results suggest that increases to the OV infection rate can lead to the success of this combination therapy within a year. The model developed here can continue to be improved as more data become available, allowing for regimen-tailoring via virtual clinical trials, ultimately shepherding effective regimens into trials.

## Introduction

Ovarian granulosa cell tumors (GCTs) account for 5% of all ovarian cancers and are known for their late and deadly recurrence, with around 80% of relapses being fatal.^1,2^ GCTs can arise at any age but usually appear in women of reproductive or postmenopausal age, and present themselves alongside menstrual irregularities, uterine bleeding and abdominal discomfort.^3–5^ Although over 80% of diagnoses are stage 1 or 2,^5,6^ the 5 and 10-year survival rates decrease quickly depending on the stage of diagnosis. The primary treatment for GCTs consists of surgical removal of the tumor,^7–9^ but chemotherapy is still used to treat ovarian GCTs^1,10^ despite the fact that a survival advantage has yet to be demonstrated.^11^ GCTs highly recurrent nature and the lack of definitive treatment highlights the critical need for novel therapeutic strategies.^10^

New approaches currently under investigation for the treatment of GCT include combining chemotherapeutic agents, such as carboplatin and paclitaxel, since certain combinations have proven effective when single therapies were ineffective.^12^ Planned properly, combination treatment can create a synergistic or additive effect thereby improving treatment efficacy by targeting different channels or therapeutic targets. They can also help overcome shortcomings and risks from conventional chemotherapy, although side-effects improve in some cases and worsen in others.^13^ In this vein, tumor necrosis factor related apoptosis-inducing ligand (TRAIL) is a prime target as a novel cancer therapy due to its selective targeting of cancer cells,^14^ low toxicity, and high tolerance in patients.^15–17^ TRAIL binds to death receptors 4 and 5 triggering the extrinsic pathway inducing apoptosis through the activation of caspase 3.^18^ TRAIL monotherapies have been less successful, producing only a single promising molecule, mapatumumab, in a non-Hodgkin lymphoma trial.^19^ Nonetheless, combination therapies involving TRAIL show potential in colorectal cancer,^20^ non-Hodgkin lymphoma,^21^ and even in ovarian cancer, where the combination of TRAIL with a chemotherapeutic agent has shown the potential to overcome therapeutic resistance.^22^

With the goal of triggering and enhancing programmed cell death in GCT cells, Crosley et al.^23^ investigated the potential of several new combination chemotherapies. They hypothesized that TRAIL works synergistically in combination with a compound named procaspase activating compound 1 (PAC-1), a caspase 3 activating agent, to increase tumor cell apoptosis, and their results support the synergistic action of PAC-1 and TRAIL in GCT.^23,24^ PAC-1 is already being investigated as a potential anti-cancer therapy in gliomas in rodents, canines, and humans^25–27^ as well as osteosarcoma and lymphoma.^28^ To circumvent TRAIL’s short half-life,^29^ Crosley et al. proposed a recombinant oncolytic vaccinia virus (OV) that promotes TRAIL production. This approach has been previously investigated using an adenovirus viral construct showing promise in human breast cancer.^30^

In oncology, around 10% of novel cancer treatments that enter phase 1 clinical trials will be approved.^31,32^ Before entering clinical trials, molecules undergo rigorous validation through *in vitro* and *in vivo* experimentation. However, results from these experiments do not guarantee successful clinical trials since the translation from animal models to clinical trials succeeds in less than 8% of studies.^33^ In the case of GCT, no animal model exists, thus forcing a reliance on the KGN cell line to develop new therapeutic approaches to treat this tumor. In response, *in silico* models can integrate data from various studies to help predict patient responses, optimize therapeutic regimens, and virtually test a huge number of scenarios to inform clinical trial designs.^34^ Thus, the integration of mathematical and computational models is cost effective^35,36^ and helps to reduce attrition along the drug development pipeline. Such models have been used in e.g., glioblastoma,^37,38^ prostate cancer,^39^ and ovarian cancer,^40^ aiding to verify therapeutic effectiveness and illustrating the adaptability and usefulness of *in silico* models. We refer readers to studies by Altrock et al.^41^ and Anderson and Quaranta^42^ that provide detailed overviews of the use of mathematical modeling in oncology.

Here we deployed a mathematical model to understand and establish the potential efficacy of combining a TRAIL-producing OV and PAC-1, and define the strategies needed for its clinical translation. Our model integrates basic viral dynamics from the OV^43,44^ and the immune pressure on the cancer cells while also including pharmacokinetic (PK) models for TRAIL and PAC-1 and their combined pharmacodynamics (PDs).^45,46^ After model parameterization to *in vitro* data, we explored the effects of increasing tumor size and the immune response to combination therapy. We also used our model to investigate escalating PAC-1 dosing regimens based on ongoing clinical trials for oral PAC-1 for anti-cancer treatment,^25^ and to establish how regimen cycle lengths and drug PKs affect treatment outcomes. Our results suggest the combination therapy in its current form is not effective enough to kill off the tumor in an *in vivo* setting, but that improvements to the PKs of PAC-1 may be a means of mitigation. Our approach still provides a safe and cost-effective way to establish promising dosing regimens and facilitate the continued clinical development of PAC-1 plus TRAIL-producing OV treatments to improve outcomes in patients with rare GCTs.

## Results

### *Combination therapy predicted to succeed* in vitro *but fails* in vivo

**Figure 1.**
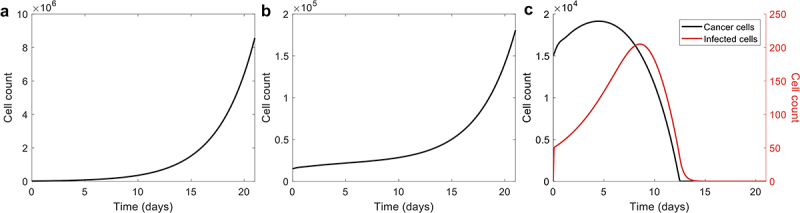
Growth of 15,000 tumour cells for different *in vitro* settings. 15,000 tumour cells growing A) in absence of the immune system, B) with the immune system, and C) with both the immune system and the oncolytic virus introduced with a MOI of 0.03. Note that the scales are different for every plot and there is no infection in A and B.

The goal of this study was to use mathematical modeling to aid in the preclinical transition of combination PAC-1 and TRAIL-producing OV to treat GCT. As there is no adequate animal model of GCT, we first used our model to translate from our *in vitro* experiment using KGN cells to *in vivo* in humans. To achieve this, we considered the model behavior in the treatment-absent context for low cell count *in vitro* where our model was fitted to the results of the proliferation assay (see Methods) seeded with 15,000 cells. We then introduced immune cells followed by the OV infection to observe their effect on a small cell count. From our initial fits (Supp. Figure S1), in absence of phagocytes and cytokine (as in our *in vitro* proliferation assay), we found exponential tumor growth. This is as expected from the *in vitro* cell seeding assay (Figure 1). However, our model predicted much slower growth once the immune system was introduced into the system (Figure 1b), consistent with the immunoediting hypothesis that the immune system initially targets tumor cells, but these latter can overcome immunosuppression.^47^ With an initial tumor size of 15,000 cells, our model then predicted that the tumor would die within 15 days after the administration of the oncolytic virus with a multiplicity of infection (MOI) of 0.03 infectious virus per cell working alongside the immune system (Figure 1c).

**Figure 2.**
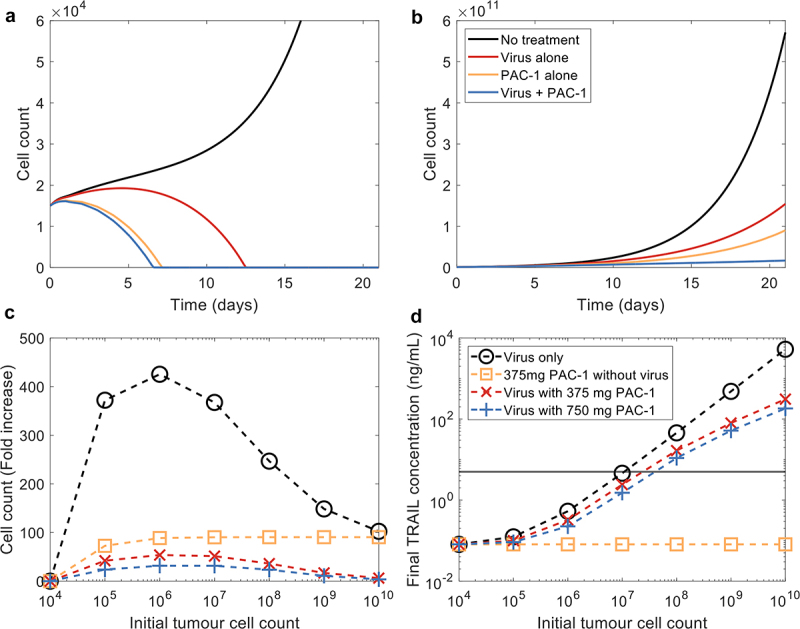
Monotherapies and combination therapy on increasing initial tumor cell count. 15,000 tumour cells growing (a) in absence of the immune system, (b) with the immune system, and (c) with both the immune system and the oncolytic virus introduced with a MOI of 0.03. Note that the scales are different for every plot and there is no infection in A and B.

We next investigated four therapy options to study the difference between monotherapies and the combination therapy over 21 days: 1) no treatment, 2) only the TRAIL-producing OV introduced on day 0 with a MOI of 0.03, 3) only daily doses of 375 mg of PAC-1, and 4) combination therapy which combines therapies 2 and 3. Figure 2a shows the effect of the various options on 15,000 cells over 21 days and both monotherapies and the combination therapy are able to kill off the tumor within two weeks. However, 15,000 cells is below the detection rate for a tumor and is too small to be relevant in humans. We, therefore, sought to increase the initial tumor cell count to explore a more realistic tumor size. To that extent, we considered an initial tumor of 1 $cm^{3}$ which amounts to $10^{9}$ initial cells, assuming a ratio of $10^{6}$ cells per mm^3 24, 48^. Applying the three therapy options to $10^{9}$ initial cells changes the previously observed dynamics and the therapies are unable to eradicate the tumour within 21 days (Figure 2b); although the growth is slowed more by the combination therapy compared to both monotherapies.

The change in dynamics from 15,000 cells to $10^{9}$ cells led us to further investigate how the combination therapy’s effectiveness changes with increasing tumor size. We decided to test four scenarios over 21 days on various tumor sizes: 1) only the OV introduced on day 0 with a MOI of 0.03 serving as our control, 2) only daily doses of 375 mg of PAC-1, 3) scenario 1 alongside daily doses of 375 mg of PAC-1 and 4) scenario 1 along daily doses of 750 mg of PAC-1. These four cases were performed on varying initial cell counts starting at $10^{5}$ and increasing 10-fold by to reach $10^{10}$ initial cells. For comparison, we analyzed the fold increase in tumor cell count by dividing the final tumor cell count by the initial cell count.

Although the fold change increases from an initial tumor cell count of $10^{5}$ to $10^{6}$, our results indicate that the effectiveness of the therapies with the OV increases as the tumour cell count increases (Figure 2c). Looking at the PAC-1 only therapy, the fold increase remains somewhat stable through the various initial tumour cell counts while the combination therapies improve with bigger cell count. This could be caused by the fact that using the same MOI for varying cell counts implies more infected cells are initially infected and would, therefore, produce more TRAIL killing off more cells over the same 21 days. To confirm this reasoning, we visualized the final TRAIL concentration for all simulations that includes the OV from Figure 2c and found that the concentration of TRAIL increased almost linearly past $10^{6}$ cells (Figure 2d). However, for smaller initial cell counts, the effect of TRAIL was found to be sub-optimal, since its final concentration after 21 days was predicted to be below TRAIL’s EC50 (horizontal black line in Figure 2d). Furthermore, daily doses of PAC-1 were predicted to reduce the number of tumor cells able to be infected, further impacting the final concentration of TRAIL for smaller tumors. Therefore, our simulations show that between $10^{7}$ and $10^{8}$ initial cells are necessary to reach the half effect concentration for TRAIL after 21 days and assure its half-maximal effect on the tumor. Together, these results (Figure 2) provide fundamental information that should guide further *in vivo* experimentation as they suggest that the OV can only drive tumor growth reduction to a certain point, and that the addition of PAC-1 improves the OV monotherapy. In its current form, our model predicts that the combination therapy is unable to eradicate realistically sized human tumors.

### Sensitivity analysis

Given these results, we wondered whether we could optimize therapeutic schedules to provide more benefit with respect to tumor reduction. Before carrying out this optimization, we wanted to assess the impact of key parameters on our model’s predictions. Thus, we performed a local sensitivity analysis to explore parameter changes from −50% to + 50% of baseline. We compared the predicted final tumor size to controls (i.e., simulations using our baseline parameters). We ran every simulation over 21 days with daily doses of 375 mg of PAC-1 and an initial MOI of 0.03 on $10^{9}$ tumor cells.

**Figure 3.**
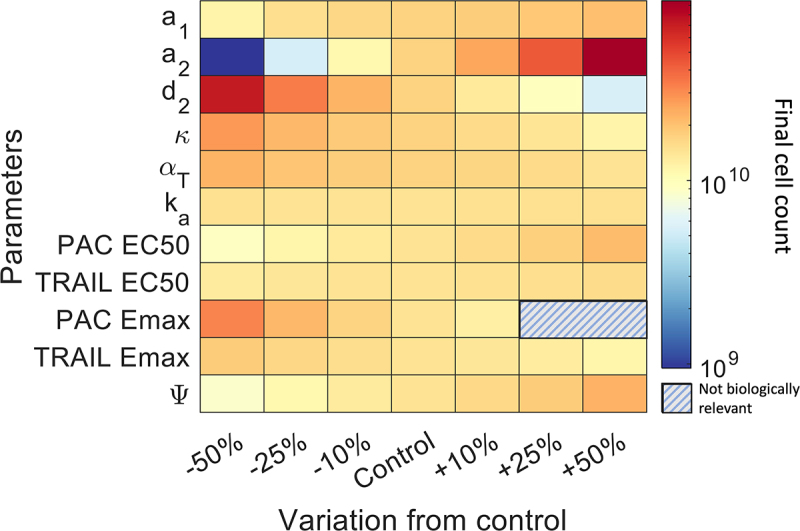
Sensitivity analysis for various parameters of the model. We considered variations from -50% to+50%. Final cell count after 7 days of combination therapy on $10^{9}$ initial cells with daily doses of 300mg of PAC-1. NaN values represent $Emax_{\;}$ values greater than 1.

This sensitivity analysis demonstrates that only a few key parameters affect the model’s output (Figure 3). These include the growth parameter $a_{2}$ and the death rate of tumour cells $d_{2}$, both of which have a direct effect on the growth of the tumour. Interestingly, $a_{1}$, which controls quiescent cell growth, showed little sensitivity to parameter modulations. This result was also replicated in the model’s PD parameters, where we found that the PAC-1 PD showed more impact than the TRAIL PD parameters, but ultimately most were nonsensitive parameters. Thus, our sensitivity analysis suggests that in addition to the initial tumor size, the stage (i.e., the growth rate) of the tumor is a key biomarker of combination treatment efficacy.

### Daily doses of PAC-1 are necessary to balance toxicity and effectiveness

Planning combination therapies for trials and optimizing regimens can be quite complex, given the large number of possible schedules. To identify schedules likely to provide the most benefit to patients based on our previous results, we began by simulating the PAC-1 treatment plan from Danciu et al.^49^ which administered PAC-1 on 28-days cycles (21 days of 75 mg-1000 mg daily oral doses of PAC-1 with 7 days of rest). In our simulations, we introduced the OV at the beginning of the first cycle on day 0 (dose with a MOI of 0.03 on $10^{9}$ initial tumour cells). We then simulated the model for 21 days and studied escalating daily doses of PAC-1 to investigate whether combination therapy with OV could reduce the overall amount of PAC-1 necessary to provide the same anti-tumoral benefit, thus minimizing a patient’s drug exposure relative to the comparable regimens studied in Danciu et al..^49^ We therefore considered two key factors: 1) is it necessary to always administer the maximal possible dose? And 2) is it necessary to administer PAC-1 every day?

**Figure 4.**
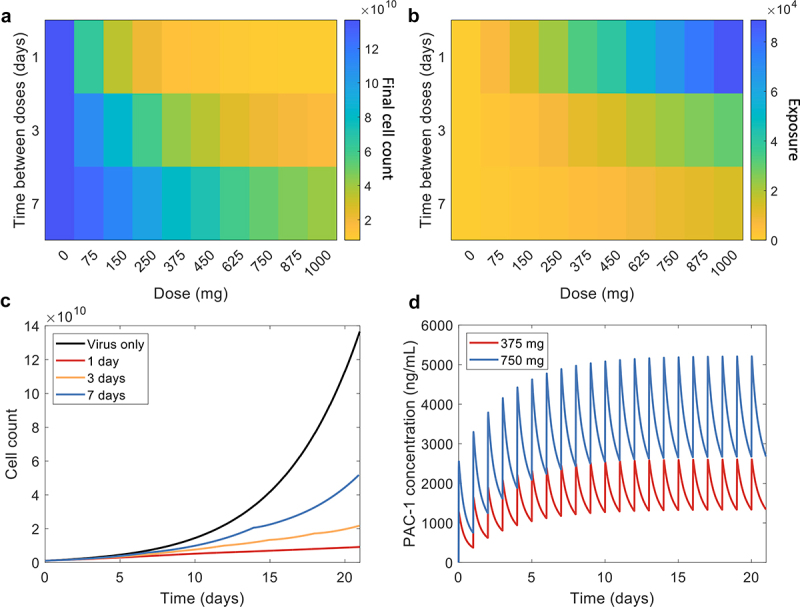
PAC-1 dose optimization using increasing time between doses. Varying time between doses and dosages for oral PAC-1. (a) Final tumor cell counts for every simulation with $10^{9}$ initial tumor cells. (b) Exposure to PAC-1 for every simulation. (c) Tumor cell count for the virus only simulation, the combination therapy using 750mg dose of PAC-1 given daily, every 3 days and every 7 days. (d) PAC-1 concentrations for 21 daily doses of 375mg and 750mg.

Our result showed that there is little benefit in giving a daily dose of PAC-1 higher than 375 mg and, further, there was no benefit to daily doses above than 750 mg (Figure 4a). With respect to daily dosing, our model predicted that waiting to give a dose of PAC-1 every 3 days or 7 days did not slow tumor growth as much as daily PAC-1 doses. This is observed particularly for e.g., doses of 750 mg, where the difference in tumor growth is clearly seen over different dosing frequencies (Figure 4c). As expected, by comparing drug exposures (calculated as the area under the curve on the concentration plot of PAC-1) of the different regimens, 1, we found that daily doses greatly increased drug exposure (Figure 4b). However, as the results of Danciu et al.^49^ did not show major toxicity or adverse effect from daily doses of 750 mg of PAC-1, the toxicity of daily doses in this range (i.e., below 1000 mg) is likely not a significant factor needing consideration going forward into trials.

We also tested giving the same total amount of 2100 mg of PAC-1 by distributing the total dose over 21 days. This translated to 21 daily doses of 100 mg, 7 doses of 300 mg every 3 days and 3 doses of 700 mg given weekly. We found that distributing the total dose gave roughly the same final tumor cell count and exposure across all three simulations but did not provide improvements over the results discussed in Figure 4 (Supp Figure S5). However, this approach to dose distribution was not found to be sufficiently effective for doses of PAC-1 below 375 mg (i.e., a total administration of 375 × 21 = 7875 mg over 21 days). Distributing 7875 mg over 21 days would translate to doses of 1125 mg every 3 days or 2625 mg once a week. The potential adverse effects of such high doses of PAC-1 are unknown. Given the results shown Figure 4, daily dosages above 375 mg were determined to be necessary, implying that even higher doses would be required if deploying this distribution strategy. Thus, we concluded that daily doses of at least 375 mg/day is the only predicted viable option for effective combination therapy.

### Increased OV infection rate leads to successful treatment outcome

**Figure 5.**
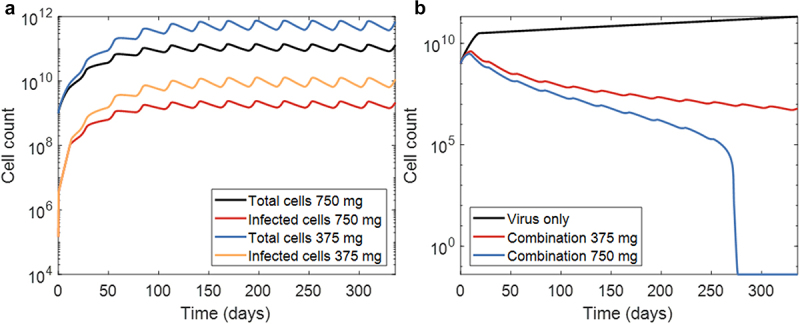
Long-term treatment using combination therapy. (a) Total tumor cell count and infected cell count for the combination therapies with either 375 or 750mg of PAC-1. (b) Therapies with infection rate 5 times the current value. Tumor cell counts below 0.01 were set to 0.01 for ease of computation but a tumor cell count below a value of 1 should be considered 0.

We next focused on long-term treatment (~1 year) outcomes. For this, we considered twelve 28-day cycles with oral PAC-1 (either 375 mg or 750 mg doses) administered daily over the first 21 days of each cycle. As before, the OV was introduced on day 0 at a MOI of 0.03 with no other OV dose is added in further cycles since no effect was seen from booster doses (data not shown). We found that the growth of the tumor was not impeded by this treatment, though the tumor cell population seemed to reach an equilibrium determined by the dose of PAC-1 (Figure 5a).

Following the sensitivity analysis from Figure 3, the viral parameter κ representing the infection rate was shown to have a minor effect on model outcomes (but was ultimately not as sensitive as other tumour growth parameters). However, as we cannot modify tumour growth rates but could potentially alter OV infection rates, we tested whether modifications to the oncolytic viral construct could provide increased benefits. Thus, we increased κ to 5 times its baseline value and simulated it alone and in combination with both doses of daily oral PAC-1. On its own the new virus remained unable to clear the $10^{9}$ initial tumour cells. However, in combination with 375 mg of daily oral PAC-1 over 21 days (28-day cycles), our model predicted tumour reductions in the magnitude of 1000-times over OV therapy on its own. Excitingly, in combination with 750 mg doses of PAC-1, the combination therapy was predicted to remove the tumor within a year (Figure 5b). Together, these results highlight the clinical potential of combination PAC-1 with a TRAIL-producing oncolytic virus.

## Discussion

Transitioning from laboratory to clinical trials to the market is complex. Results from *in vitro* experiments do not always replicate in mice models and the same is true in humans. These failures cause high costs in terms of time, research and development expenses, and patient investments. The development of mathematical models alongside preclinical experimental work can provide insights into the potential of novel cancer therapies, determine optimal treatment schedules to move forward into trials, and inform on needed improvements to drug formulations, helping to accelerate drug development.

To this end, we developed a mathematical model to investigate the *in vivo* potential of combination PAC-1 and a TRAIL-producing OV to treat granulosa cell tumor of the ovary. This model includes the viral dynamics of the OV as well as the PK/PD of both PAC-1 and (endogenous and exogenous) TRAIL. Our model corroborates the success of this combination when considering low cell counts, predicting that the joint tumor suppression effects derived from OV infection and the immune system can kill off a small tumor cell population. However, our model predicts that it is likely that, in humans, the current combination therapy is unable to stop the growth of the tumor cell population. Here, it is worth noting that the lack of animal model forced us to rely on *in vitro* data for the tumor growth parameters and, according to our sensitivity analysis, the model’s predictions are particularly impacted by the parameters governing growth. Therefore, our model would benefit greatly from *in vivo* animal data to improve the accuracy of our results.

Following the results from Danciu et al., our simulations corroborate the fact that daily doses of 750 mg were optimal in the use of oral doses of PAC-1 although, a beneficial effect was also observed for dosages above 375 mg. However, due to its PKs, the concentration of PAC-1 can accumulate over multiple daily doses and reach a steady-state concentration, and we found that the drug exposure could rise quickly with increased dosages. To understand potential toxicities of the higher daily dosing schedule, we calculated the area under the PAC-1 concentration curve and compared its exposure with other regimens with longer periods between PAC-1 doses. Although we found that total exposure was much higher for daily doses, previous clinical results from Danciu et al. did not suggest dangerous toxicities at these doses. Thus, we conclude that toxicity with doses within this range is a potentially limited factor to the continued development of the combination regimen studied here. It is worth mentioning that the different doses of PAC-1 used in our simulations do not consider a patient’s weight or body surface area. A proper analysis of the toxicity would require a virtual cohort with such parameters, but this is not the aim of this paper.

To study the long-term effects of treatment, we simulated 28-day cycles with 21-day “on” cycles of oral PAC-1. For this regimen, we observed that a single dose of the OV introduced on day 0 can sustain the tumor infection. We found that the tumor cell population grew rapidly over the first 4 cycles before stabilizing toward a fixed population size that depended on the dose size of PAC-1. Even at higher 750 mg doses, the combination therapy was unable to kill off the tumor. To test whether tumor eradication could be possible with this combined therapy, we tested an infection rate increased to be 5-times the baseline estimate in our model. Excitingly, in this scenario our model predicted that combination therapy of TRAIL-producing OV and daily doses of 750 mg of PAC-1 (21-days on, 7-days off) can eradicate the tumor within a year. This prediction remains theoretical but nonetheless demonstrates the additive or synergistic potential of this combination since the OV or PAC-1 alone were not able to completely kill the tumor. However, it is necessary to mention that the OV dynamics are not behaving in the way we would expect . A single dose of a vaccinia OV should realistically not last a whole year and multiple booster doses would be expected since previous experiments and clinical trials using oncolytic vaccinia virus required multiple doses.^50,51^ To properly investigate long-term behavior, our model would require the inclusion of adaptive immunity to depict the tumor eradication by the immune system more accurately as well as improving virus clearance from the system, which is the subject of ongoing research. As before, with more appropriate data, we could observe more realistic results but for the purpose of our model, our results still confirm the potential of the combination therapy of a TRAIL-producing OV and daily doses of oral PAC-1.

Before such a therapy moves toward clinical trials, much more work needs to be done to improve upon the framework we built. As mentioned earlier, the lack of a mouse model and *in vivo* data may significantly affect the accuracy of our results. Furthermore, the parameters for the viral dynamics were not re-estimated here, though they were obtained after extensive parameterization to an OV using a similar virus. Future studies will focus on the viral dynamics of our particular OV construct. Despite these limitations, our model can be easily and rapidly adapted to accommodate new data and answer questions arising in the continued development of combination TRAIL-producing OV and PAC-1. Our framework is therefore a good example of the continued integration of mathematical modeling with preclinical drug development pipelines.

## Materials and methods

### Proliferation assay

The human GCT cell line (KGN (Riken Biosciences)) was cultured in Dulbecco’s Modified Eagle’s Medium/Nutrient Mixture F12 (DMEM/F12, Sigma-Aldrich) with 5% fetal bovine serum (FBS, Gibco). On day −1 (D-1) cells were seeded in 24-well plates where wells received 15,000 cells. On D0 through D6, duplicate wells were trypsinized, stained with Trypan blue (ThermoFisher # 15250061), loaded on a hemacytometer, and counted under a microscope at low magnification. The assay was repeated in triplicate.

### Mathematical model of combination TRAIL-producing OV and PAC-1 to treat GCT

**Figure 6.**
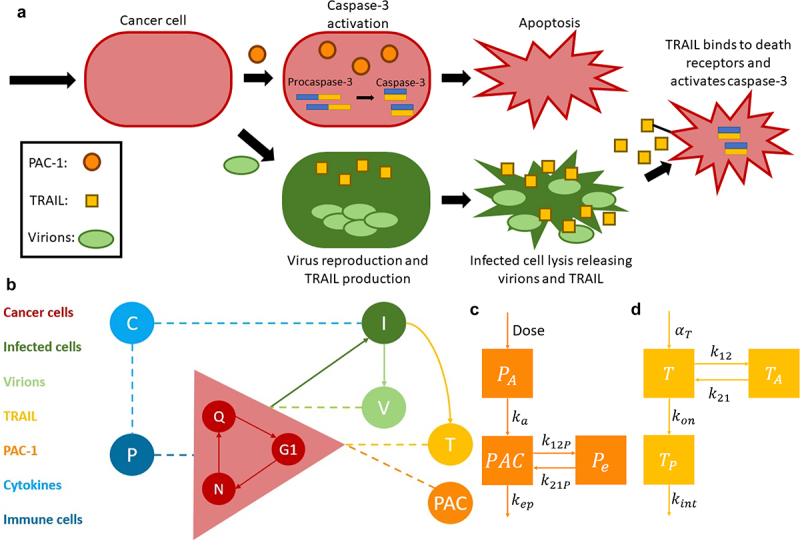
Biological representation and mathematical translation of the combination therapy of a TRAIL-producing OV with PAC-1. (a) conceptual model of combination PAC-1 and TRAIL-producing OV. PAC-1 enters cancer cells activating caspase-3 initiating cellular apoptosis. OV virions enter cancer cells and begin replication and TRAIL production. Upon lysis, TRAIL is released, alongside newly formed virions, to bind to DR4 and DR5 of other cancer cells. The binding of TRAIL initiates the apoptotic pathways leading to caspase-3 activation and apoptosis. (b) mathematical model schematic. The model components include compartments for the tumor cells going through the cell cycle (*Q*, *G*_1_, *N*) and the viral dynamic of virion particles (*V*) creating infected cells (*I*) from cancer cells. The immune cells (*P*) are alerted by cytokines (*C*) from the localized infection while the combination therapy of TRAIL and PAC-1 induces apoptosis in cancer cells. (c) schematic of the pharmacokinetics model for PAC-1 including the GI-tract compartment (*P_A_*) the plasma concentration (*PAC*) and the additional compartment (*P_e_*). (d) schematic of the TRAIL pharmacokinetics model describing free TRAIL (*T*), TRAIL complex (*T_P_*) and TRAIL in peripheral tissues (T_A_).

We developed a mathematical model of combination TRAIL-producing OV and PAC-1 based on our previous work modeling combination virotherapies and the synergy between TRAIL and PAC-1.^52–54^ Because OVs depend on cellular division for the continued production of virions, our model describes tumor growth through each stage of the cell cycle, i.e., quiescent through $G_{1}$ and active phases. In the following sections, we describe each of the model components from the tumor growth to the PK and PD aspects of the model. A graphical representation for the biological concept of this combination therapy can be found in Figure 6a followed by a complete model schematic in Figure 6b–d.

#### Mathematical model of GCT tumour growth

Let $Q\left(t\right)$ represent the number of quiescent tumour cells, $G_{1}\left(t\right)$ the number of cells in the $G_{1}$ phase, and $A_{i}\left(t\right)$ the ith compartment of the active phases of the cell cycle (with $N\left(t\right)$ the sum of all the active compartments, where i=1:n). As in Cassidy and Craig and Jenner et al.,^52–54^ we account for a variable duration of these phases using the transformation of a distributed delay into ordinary differential equations (ODEs) via the linear chain technique.^53–55^ Quiescent cells enter $G_{1}$ at rate $a_{1}$, enter the active phases at rate $a_{2}$, and undergo apoptosis at rate $d_{2}$. The cells then enter the first active compartment $A_{1}\left(t\right)$ at rate $a_{2}$ before transiting towards other active compartments $A_{i}\left(t\right)$at rate $k_{tr}$. Finally, throughout those active compartments, $A_{i}$, the cells can die at rate $d_{3}$. The system of equations below represents these assumptions:

(1) $$\frac{dQ}{dt}=2k_{tr}A_{j}\left(t\right)-a_{1}Q\left(t\right)$$

(2) $$\frac{dG_{1}}{dt}=a_{1}Q\left(t\right)-\left[a_{2}+d_{2}\right]G_{1}\left(t\right)$$

(3) $$\frac{dA_{1}}{dt}=a_{2}G_{1}\left(t\right)-k_{tr}A_{1}\left(t\right)-d_{3}A_{1}\left(t\right)$$

(4) $$\frac{dA_{i}}{dt}=k_{tr}\left(A_{i-1}\left(t\right)-A_{i}\left(t\right)\right)-d_{3}A_{i}\left(t\right)$$

(5) $$\frac{dN}{dt}=a_{2}G_{1}\left(t\right)-d_{3}N\left(t\right)-\frac{k_{tr}}{a_{2}}A_{n}\left(t\right)$$

#### Modeling OV infection

We modeled the kinetics of the oncolytic virus based on a previously established viral dynamics model.^43^ In our model, infected cells ($I\left(t\right)$) are produced through mass-action contact dynamics between viral particles $V\left(t\right)$ and cells in $G_{1}$ and the active phases of the cell cycle ($N\left(t\right)$) at rate $\kappa \eta \left(V\left(t\right)\right)$ which also considers the virions half effect concentration $\eta_{1/2}$:

(6) $$\kappa \eta \left(V\left(t\right)\right)=\kappa \frac{V\left(t\right)}{\eta_{1/2}+V\left(t\right)}$$

(7) $$\frac{dI}{dt}=\kappa \eta \left(V\left(t\right)\right)\left(G_{1}\left(t\right)+N\left(t\right)\right)-\delta I$$

(8) $$\frac{dV}{dt}=\alpha \delta I-\omega V-\kappa \eta \left(V\left(t\right)\right)\left(G_{1}\left(t\right)+N\left(t\right)\right)$$

Infected cells then undergo lysis at rate δ, releasing viral particles at rate α and these virions decay exponentially at rate ω. We assume that quiescent cells are unable to actively produce virus and hence are not modeled as becoming infected in this work. The introduction of the OV was accomplished as an initial condition on the number of virions $V\left(0\right)$.

#### Mathematical model of tumour-innate immune interactions

Oncolytic viruses play a dual role in that they infect and lyse tumor cells while simultaneously eliciting a strong anti-tumoral innate immune response.^56,57^ The immune system modeled here was parameterized in Jenner et al.^52^ and models the innate immune pressure through the functions $\psi_{Q}\left(t\right)$ and $\psi_{G}\left(t\right)$ that account for the population of phagocytes ($P\left(t\right)$), the contact rate between these cells and tumour cells ($k_{p}$), and the phagocyte cell digestion constants ($k_{Q}$ and $k_{s}$). For complete details on the sensitivity analysis of this model, see Jenner et al.^52^

To account for immune stimulation, we also modeled the immune response induced by phagocytes stimulated and attracted to the site of oncolytic virus infection through cytokine signaling. Cytokine ($C\left(t\right)$) is produced at rate $C_{prod}$ depending on the number of infected cells ($I\left(t\right)$) and is cleared at rate $k_{elim}$. Cytokine/phagocyte feedback regulates the number of tumor-specific phagocytes at rate $\phi \left(C\left(t\right)\right)$, and these immune cells die at rate $\gamma_{P}$.

(9) $$\psi_{Q}\left(t\right)=\frac{k_{p}P\left(t\right)}{1+k_{Q}Q\left(t\right)}$$

(10) $$\psi_{G}\left(t\right)=\frac{k_{p}P\left(t\right)}{1+k_{s}G_{1}\left(t\right)}$$

(11) $$C_{prod}\left(t\right)=C_{prod}^{*}+\left(C_{prod}^{max}-C_{prod}^{*}\right)\frac{\delta I+\Psi \left(t\right)}{\Psi_{1/2}+\delta I+\Psi \left(t\right)}$$

(12) $$\Psi \left(t\right)=\psi_{G}\left(t\right)\cdot \left(G_{1}\left(t\right)+N\left(t\right)\right)+\psi_{Q}\left(t\right)\cdot Q\left(t\right)$$

(13) $$\phi \left(C\left(t\right)\right)=\frac{k_{cp}C\left(t\right)}{C_{1/2}+C\left(t\right)}$$

(14) $$\frac{dC}{dt}=C_{prod}\left(t\right)-k_{elim}C\left(t\right)$$

(15) $$\frac{dP}{dt}=\phi \left(C\left(t\right)\right)-\gamma_{p}P\left(t\right)$$

#### Modeling the pharmacokinetics of PAC-1 and TRAIL

We modeled PAC-1 pharmacokinetics according to a two-compartment model with oral administration (Figure 6c), based on a phase 1 clinical trial.^49^ The dose was modeled as first entering the GI tract ($P_{A}\left(t\right)$) before being absorbed into the plasma ($PAC\left(t\right)$) at rate $k_{a}$. Once in the plasma, PAC-1 is eliminated at rate $k_{ep}$ or can be exchanged with the peripheral compartment $P_{e}\left(t\right)$ using the transit parameters $k_{12P}$ and $k_{21P}$. It is important to note that though doses are referenced throughout in units of mg, the units of the $P_{A}$ compartment are in ng and both other compartments are in ng/mL.

(16) $$\frac{dP_{A}}{dt}=-k_{a}P_{A}\left(t\right)$$

(17) $$\frac{dPAC}{dt}=\frac{k_{a}P_{A}\left(t\right)}{V_{PAC}}-k_{ep}PAC\left(t\right)-k_{12P}PAC\left(t\right)+k_{21P}P_{e}\left(t\right)$$

(18) $$\frac{dP_{e}}{dt}=k_{12P}PAC\left(t\right)-k_{21P}P_{e}\left(t\right)$$

TRAIL PKs were modeled to follow an irreversible binding target-mediated drug disposition (TMDD) model with a constant number of receptors ($R_{0}$)^58,59^ (Figure 6d). This model considers three compartments: free ligand TRAIL ($T\left(t\right)$), the complex of receptor-bound TRAIL ($T_{P}\left(t\right)$), and the amount of ligand in the peripheral tissue ($T_{A}\left(t\right)$). TRAIL was produced at rate $\alpha_{T}$ from the lysis of infected cells and intrinsically at a constant rate $T_{prod}$. TRAIL was modeled as being eliminated at rate $k_{el}$. It binds to the death receptors 4 and 5 to form a complex at rate $k_{on}$ and transits to and from the ligand compartment ($T_{A}\left(t\right)$) at rates $k_{12}$ and $k_{21}$, respectively. Once the complex is formed, it is degraded at rate $k_{int}$.

(19) $$\frac{dT}{dt}=\alpha_{T}\delta I-k_{el}T-k_{on}T\left(R_{0}-T_{P}\right)-k_{12}T+k_{21}\frac{T_{A}}{V_{T}}+T_{prod}$$

(20) $$\frac{dT_{P}}{dt}=k_{on}R_{0}T-\left(k_{int}+k_{on}T\right)T_{P}$$

(21) $$\frac{dT_{A}}{dt}=k_{12}TV_{T}-k_{21}T_{A}$$

#### Pharmacodynamics of the combination therapy

To model the combined effects of PAC-1 plus TRAIL-producing OV, we adopted the PD function from Chakraborty and Jusko originally developed to describe the pharmacodynamics of interleukin-10 and prednisolone.^60^ This model describes the PD effect of two molecules (here TRAIL and PAC-1) alone and in combination, based on the well-known Hill function.^61^ Each molecule is described through three parameters: the drug concentration to achieve 50% of the maximal effect (EC50), the maximal effect (Emax) and the Hill coefficient (γ). In combination, a potency term (Ψ), accounting for the combination effect between the two molecules is introduced and describes synergistic (Ψ<1), additive (Ψ=1), or antagonistic (Ψ>1) effects. Furthermore, since TRAIL is naturally found in the body, we consider the difference in TRAIL concentration from the homeostatic concentration $T^{*}$. The parameters for this equation were previously fit for the PAC-1 and TRAIL synergy.^23^

(22) $$\begin{array}{rl} & E\left(PAC,T\right)=\\ & \frac{\left[\begin{array}{c} E_{max,PAC}{\left(\frac{PAC}{\Psi \cdot EC50_{PAC}}\right)}^{\gamma_{PAC}}+E_{max,TRAIL}{\left(\frac{T-T^{*}}{\Psi \cdot EC50_{TRAIL}}\right)}^{\gamma_{TRAIL}}\\ +\left(E_{max,PAC}+E_{max,TRAIL}-E_{max,PAC}E_{max,TRAIL}\right)\\ \times {\left(\frac{PAC}{\Psi \cdot EC50_{PAC}}\right)}^{\gamma_{PAC}}{\left(\frac{T-T^{*}}{\Psi \cdot EC50_{TRAIL}}\right)}^{\gamma_{TRAIL}} \end{array}\right]}{\left[\begin{array}{c} 1+{\left(\frac{PAC}{\Psi \times EC50_{PAC}}\right)}^{\gamma_{PAC}}+{\left(\frac{T-T^{*}}{\Psi \times EC50_{TRAIL}}\right)}^{\gamma_{TRAIL}}\\ +{\left(\frac{PAC}{\Psi \times EC50_{PAC}}\right)}^{\gamma_{PAC}}{\left(\frac{T-T^{*}}{\Psi \times EC50_{TRAIL}}\right)}^{\gamma_{TRAIL}} \end{array}\right]} \end{array}$$

#### Complete model

Together, these sub models describe tumor growth affected by immune cells and infection by the OV, as well as the joint effects of PAC-1 and TRAIL:

(23) $$\frac{dQ}{dt}=2k_{tr}A_{j}\left(t\right)-a_{1}Q\left(t\right)-\psi_{Q}\left(t\right)Q\left(t\right)$$

(24) $$\frac{dG_{1}}{dt}=a_{1}Q\left(t\right)-\left[a_{2}+d_{2}\left(1+E\left(PAC,T\right)\right)+\kappa \eta \left(V\left(t\right)\right)+\psi_{G}\left(t\right)\right]G_{1}\left(t\right)$$

(25) $$\frac{dA_{1}}{dt}=a_{2}G_{1}\left(t\right)-k_{tr}A_{1}\left(t\right)-\left[d_{3}\left(1+E\left(PAC,T\right)\right)+\kappa \eta \left(V\left(t\right)\right)+\psi_{G}\left(t\right)\right]A_{1}\left(t\right)$$

(26) $$\frac{dA_{i}}{dt}=k_{tr}\left(A_{i-1}\left(t\right)-A_{i}\left(t\right)\right)-\left[d_{3}\left(1+E\left(PAC,T\right)\right)+\kappa \eta \left(V\left(t\right)\right)+\psi_{G}\left(t\right)\right]A_{i}\left(t\right)$$

(27) $$\frac{dN}{dt}=a_{2}G_{1}\left(t\right)-\left[d_{3}\left(1+E\left(PAC,T\right)\right)+\kappa \eta \left(V\left(t\right)\right)+\psi_{G}\left(t\right)\right]N\left(t\right)-\frac{k_{tr}}{a_{2}}A_{n}\left(t\right)$$

(28) $$\frac{dI}{dt}=\kappa \eta \left(V\left(t\right)\right)\left(G_{1}\left(t\right)+N\left(t\right)\right)-\delta I$$

(29) $$\frac{dV}{dt}=\alpha \delta I-\omega V-\kappa \eta \left(V\left(t\right)\right)\left(G_{1}\left(t\right)+N\left(t\right)\right)$$

(30) $$\frac{dC}{dt}=C_{prod}\left(t\right)-k_{elim}C\left(t\right)$$

(31) $$\frac{dP}{dt}=\phi \left(C\left(t\right)\right)-\gamma_{p}P\left(t\right)$$

(32) $$\frac{dP_{A}}{dt}=-k_{a}P_{A}\left(t\right)$$

(33) $$\frac{dPAC}{dt}=\frac{k_{a}P_{A}\left(t\right)}{V_{PAC}}-k_{ep}PAC\left(t\right)-k_{12P}PAC\left(t\right)+k_{21P}P_{e}\left(t\right)$$

(34) $$\frac{dP_{e}}{dt}=k_{12P}PAC\left(t\right)-k_{21P}P_{e}\left(t\right)$$

(35) $$\frac{dT}{dt}=\alpha_{T}\delta I-k_{el}T-k_{on}T\left(R_{0}-T_{P}\right)-k_{12}T+k_{21}\frac{T_{A}}{V_{T}}+T_{prod}$$

(36) $$\frac{dT_{P}}{dt}=k_{on}R_{0}T-\left(k_{int}+k_{on}T\right)T_{P}$$

(37) $$\frac{dT_{A}}{dt}=k_{12}TV_{T}-k_{21}T_{A}$$

### Parameter estimation

To parameterize the model, we used a stepwise approach beginning with a smaller set of equations to replicate our *in vitro* proliferation assay results. This sub model only considers the cancer cell growth (involving only Q, $G_{1},A_{i}$ and N), as described in the section *Mathematical model of GCT tumour growth* including five parameters, namely $a_{1}$, $a_{2}$, $d_{2}$, $d_{3}$ and $k_{tr}$. However, the transformation of this sub model by Cassidy, Craig and Jenner et al.^52–54^ into ODEs leads to parameters $d_{3}$ and $k_{tr}$ being calculated based on the number of transit compartments n and the intermitotic time. Both these values are calculated using the $a_{2}$ parameter implying that we ultimately only needed to fit $a_{1}$, $a_{2}$ and $d_{2}$. These three parameters thus form our parameter space θ (see Figure S1). The total number of cells ($y\left(t\right)=Q+G_{1}+N$) in the model were compared to the average cell count ($\overset{ˉ}{x}$) from the three replicates in the wells using the *lsqnonlin* function in MATLAB.^62^ This non-linear least-square method minimizes the square of the Euclidean norm of the vector function f:

(38) $$\min_{\theta }||f\left(\theta \right)|{|}_{2}^{2}=\min_{\theta }\sum {\left(f_{i}\left(\theta \right)\right)}^{2}$$

(39) $$f\left(\theta \right)=\left[\begin{array}{cc} \begin{array}{cc} y\left(t_{1},\theta \right)-{\overset{ˉ}{x}}_{1}, & y\left(t_{2},\theta \right)-{\overset{ˉ}{x}}_{2}, \end{array} & \begin{array}{cc} \cdots & y\left(t_{n},\theta \right)-{\overset{ˉ}{x}}_{n} \end{array} \end{array}\right]$$

In our case, the elements of f were calculated using the difference between the fit at time $t_{i}$ for a given parameter set θ, i.e., $y\left(t_{i},\theta \right)$, and the average of the data at the same time, ${\overset{ˉ}{x}}_{i}$. It is also important to note that we disregarded the last data point from our proliferation assay results as it was lower than the previous data point at Day 6, suggesting cells in the dish were dying, which was not accounted for in our model. Viral parameters and immune system parameters were taken from Jenner et al.^52^ and can be found in the associated Supplementary Information. As the OV in this work is based on the vaccinia virus, only the parameters associated with that OV were used.

The rate of TRAIL production ($\alpha_{T}$) was estimated using *lsqnonlin* in MATLAB from digitized data from Oh et al.^63^ (see Figure S2). Assuming exponential production of TRAIL, we compared the final production of TRAIL at the end of the hypoxia experiment in Oh et al..^63^ The basal TRAIL concentration ($T^{*}$) was estimated from Xiang et al.,^64^ and the natural production of TRAIL by the body ($T_{prod}$) was calculated directly assuming homeostasis for $T\left(t\right)$ in Equations 23-37 to ensure model stability in the absence of the OV.

All other TRAIL model parameters were estimated using digitized data from Kelley et al.^65^ (Figure S3). For this, we used the in-built Monolix target-mediated drug disposition model with a constant R0 and irreversible binding. Monolix is software suite dedicated to pharmacometrics-oriented modeling and simulation for drug development. Monolix uses nonlinear mixed effect (NLME) models to estimate parameters while accounting for interindividual variability (IIV). For a given parameter P, the model considers the population average ${\overset{ˉ}{P}}_{pop}$ as well as the IIV represented as a normally distributed variable $\eta_{P}$ of mean 0 and variance $\omega_{p}^{2}$:

(40) $$P={\overset{ˉ}{P}}_{pop}\cdot e^{\eta_{p}}$$

Through stochastic approximation expectation-maximization, Monolix estimates PK parameters for a specific structural model (either in-built or user-provided) based on the relevant data. The documentation for Monolix as well as its library of integrated models can be found at https://monolix.lixoft.com/.

To estimate the models in the PAC-1 pharmacokinetic model, we digitized data from Lucas et al.^66^ and used the in-built one-compartment oral PK model in Monolix, similar to the TRAIL model (see Figure S4). For both TRAIL and PAC-1 pharmacodynamics, parameters were taken directly from Cardinal et al.^24^

### Sensitivity analysis

We performed a sensitivity analysis by individually increasing or decreasing selected parameters by 10%, 25% and 50%. Parameters were selected based on their ability to greatly impact tumor growth, and include the baseline growth parameters ($a_{1}$, $a_{2}$ and $d_{2}$), the infection rate (κ), the TRAIL production from the OV ($\alpha_{T}$), the absorption rate of PAC-1 ($k_{a}$) and, finally, EC50 and Emax for both TRAIL and PAC-1 as well as their synergistic potency term (Ψ). For each parameter value, we simulated the model (Equations 23-37) with $10^{9}$ initial cells over 21 days of combination therapy and recorded the final amount of tumor, infected, and non-infected cells as outputs. Then, we compared the final number of cells with the final number of cells in the control simulation to determine the level of sensitivity of each parameter.

## Supplementary Material

SI_JLSR.docx
