# Supplementary material for: Mathematical modeling predicts pathways to successful implementation of combination TRAIL-producing oncolytic virus and PAC-1 to treat granulosa cell tumors of the ovary
Source: Cancer Biol Ther. 2023 Nov 27;24(1):2283926. doi: 10.1080/15384047.2023.2283926 (PMC10783843; doi:10.1080/15384047.2023.2283926)
Supplement: SI_JLSR.docx [file KCBT_A_2283926_SM1322.docx]

**Supplementary information to: Mathematical modelling predicts pathways to successful implementation of combination TRAIL-producing oncolytic virus and PAC-1 to treat granulosa cell tumours of the ovary**

**Justin Le Sauteur-Robitaille, Powel Crosley, Mary Hitt, Adrianne L. Jenner, Morgan Craig**

| **Parameters** | **Units** | **Descriptions** | **Values** | **Sources** |
| --- | --- | --- | --- | --- |
| $a_{1}$ | 1/day | $Q$ to $G_{1}$ rate | $3.3498$ | Fit from data |
| $a_{2}$ | 1/day | $G_{1}$ to $A_{1}$ rate | $1.44$ | Fit from data |
| $d_{2}$ | 1/day | $G_{1}$ apoptotic rate | $0.2$ | Fit from data |
| $d_{3}$ | 1/day | Active phase apoptotic rate | $0.1732$ | Calculated |
| $k_{tr}$ | 1/day | Active phase transfer rate | $8.4540$ | Calculated |
| $\kappa$ | 1/day | Virion infection rate | $0.054$ | Jenner et al.^1^ |
| $\delta$ | 1/day | Lysis rate | $2.48$ | Jenner et al.^1^ |
| $\alpha$ | Virions/cell | Burst size | $1.12$ | Jenner et al.^1^ |
| $\omega$ | 1/day | Virion decay rate | $40.3$ | Jenner et al.^1^ |
| $k_{p}$ | 1/day | Phagocyte-tumour cell contact rate | $9.23$ | Jenner et al.^1^ |
| $k_{q}$, $k_{s}$ | - | Phagocyte cell digestion constant | $0.064$ | Jenner et al.^1^ |
| $\Psi_{1/2}$ | ${10}^{10}$cells/day | Cytokine production half effect | $0.00011$ | Jenner et al.^1^ |
| $k_{cp}$ | ${10}^{10}$cells/day | Maximal immune cell production rate | $4.6754$ | Jenner et al.^1^ |
| $\eta_{1/2}$ | Virions | Virion half effect concentration | $0.51$ | Jenner et al.^1^ |
| $C_{1/2}$ | ng/mL/day | Phagocyte production half effect | $0.739$ | Jenner et al.^1^ |
| $\gamma_{P}$ | 1/day | Phagocyte death rate | $0.35$ | Jenner et al.^1^ |
| $C_{prod}^{*}$ | ng/mL/day | Homeostatic cytokine production rate | $3.9863\times{10}^{-4}$ | Jenner et al.^1^ |
| $C_{prod}^{max}$ | ng/mL/day | Maximal cytokine production rate | 1.429 | Jenner et al.^1^ |
| $k_{elim}$ | 1/day | Cytokine eliminate rate | 0.16139 | Jenner et al.^1^ |
| $j$ | - | Number of transit compartments | 6 | Calculated |
| $\tau$ | days | Expected cell cycle duration | $0.7097$ | Calculated |
| $T^{*}$ | ng/mL | Homeostatic TRAIL concentration | $0.08090$ | Xiang et al.^2^ |

**Table 1: List of parameters for the model.** Contains cell growth parameters, viral parameters and immune system parameters as well as other necessary values.

| **Parameters** | **Units** | **Descriptions** | **Values** | **Sources** |
| --- | --- | --- | --- | --- |
| $k_{a}$ | 1/day | PAC-1 oral absorption rate | $2.96$ | Fit using data from Danciu et al.^3^ |
| $V_{PAC}$ | mL | Volume of PAC-1 compartment | $3390.45$ | Fit using data from Danciu et al.^3^ |
| $k_{ep}$ | 1/day | PAC-1 elimination rate | $61.97$ | Fit using data from Danciu et al.^3^ |
| $k_{12P}$ | 1/day | Transfer rate from $PAC$ to $P_{e}$ | $183.49$ | Fit using data from Danciu et al.^3^ |
| $k_{21P}$ | 1/day | Transfer rate from $P_{e}$ to $PAC$ | $1.18$ | Fit using data from Danciu et al.^3^ |
| $\alpha_{T}$ | ng/mL/cell | TRAIL production from virus | $7.5837\times{10}^{-6}$ | Fit using data from Oh et al.^4^ |
| $k_{el}$ | 1/day | TRAIL elimination rate | 45 | Fit using data from Kelley et al.^5^ |
| $k_{on}$ | 1/day | TRAIL binding rate | 0.026 | Fit using data from Kelley et al.^5^ |
| $R_{0}$ | ng/mL | Initial bound TRAIL and receptor complex target concentration | 457.49 | Fit using data from Kelley et al.^5^ |
| $k_{12}$ | 1/day | Transfer rate from $T$ to $T_{A}$ | 11.38 | Fit using data from Kelley et al.^5^ |
| $k_{21}$ | 1/day | Transfer rate from $T_{A}$ to $T$ | 0.0043 | Fit using data from Kelley et al.^5^ |
| $V$ | mL | Volume of TRAIL main compartment | 100.04 | Fit using data from Kelley et al.^5^ |
| $k_{int}$ | 1/day | Bound TRAIL Internalization rate | 22.15 | Fit using data from Kelley et al.^5^ |

**Table 2: List of PK parameters.** Contains parameters for the PAC-1 two-compartment model and the TRAIL TMDD model.

| **Parameters** | **Units** | **Descriptions** | **Values** | **Sources** |
| --- | --- | --- | --- | --- |
| $E_{max,PAC}$ | - | Maximum efficacy of PAC-1 | $0.8764$ | Cardinal et al.^6^ |
| $E_{max,TRAIL}$ | - | Maximum efficacy of TRAIL | $0.438$ | Cardinal et al.^6^ |
| $EC{50}_{PAC}$ | ng/mL | PAC-1 half effect concentration | $1176.7$ | Calculated from Cardinal et al.^6^ |
| $EC{50}_{TRAIL}$ | ng/mL | TRAIL half effect concentration | $5$ | Cardinal et al.^6^ |
| $\gamma_{PAC}$ | - | PAC-1 hill coefficient | 1.35 | Cardinal et al.^6^ |
| $\gamma_{TRAIL}$ | - | TRAIL hill coefficient | 0.874 | Cardinal et al.^6^ |
| $\Psi$ | - | Potency | 0.8 | Cardinal et al.^6^ |

**Table 3: List of PD parameters.** Parameters necessary to the joint effect function from Eq.22.

**References**

1. Jenner, A. L., Cassidy, T., Belaid, K., Bourgeois-Daigneault, M.-C. & Craig, M. In silico trials predict that combination strategies for enhancing vesicular stomatitis oncolytic virus are determined by tumor aggressivity. *J. Immunother. Cancer* **9**, e001387 (2021).

2. Xiang, G., Zhang, J., Ling, Y. & Zhao, L. Circulating level of TRAIL concentration is positively associated with endothelial function and increased by diabetic therapy in the newly diagnosed type 2 diabetic patients. *Clin. Endocrinol. (Oxf.)* **80**, 228–234 (2014).

3. Danciu, O. C. *et al.* Phase I study of procaspase-activating compound-1 (PAC-1) in the treatment of advanced malignancies. *Br. J. Cancer* **128**, 783–792 (2023).

4. Oh, E., Hong, J., Kwon, O.-J. & Yun, C.-O. A hypoxia- and telomerase-responsive oncolytic adenovirus expressing secretable trimeric TRAIL triggers tumour-specific apoptosis and promotes viral dispersion in TRAIL-resistant glioblastoma. *Sci. Rep.* **8**, 1420 (2018).

5. Kelley, S. K. *et al.* Preclinical Studies to Predict the Disposition of Apo2L/Tumor Necrosis Factor-Related Apoptosis-Inducing Ligand in Humans: Characterization of in Vivo Efficacy, Pharmacokinetics, and Safety. *J. Pharmacol. Exp. Ther.* **299**, 31–38 (2001).

6. Cardinal, O. *et al.* Establishing combination PAC-1 and TRAIL regimens for treating ovarian cancer based on patient-specific pharmacokinetic profiles using in silico clinical trials. *Comput. Syst. Oncol.* **2**, e1035 (2022).


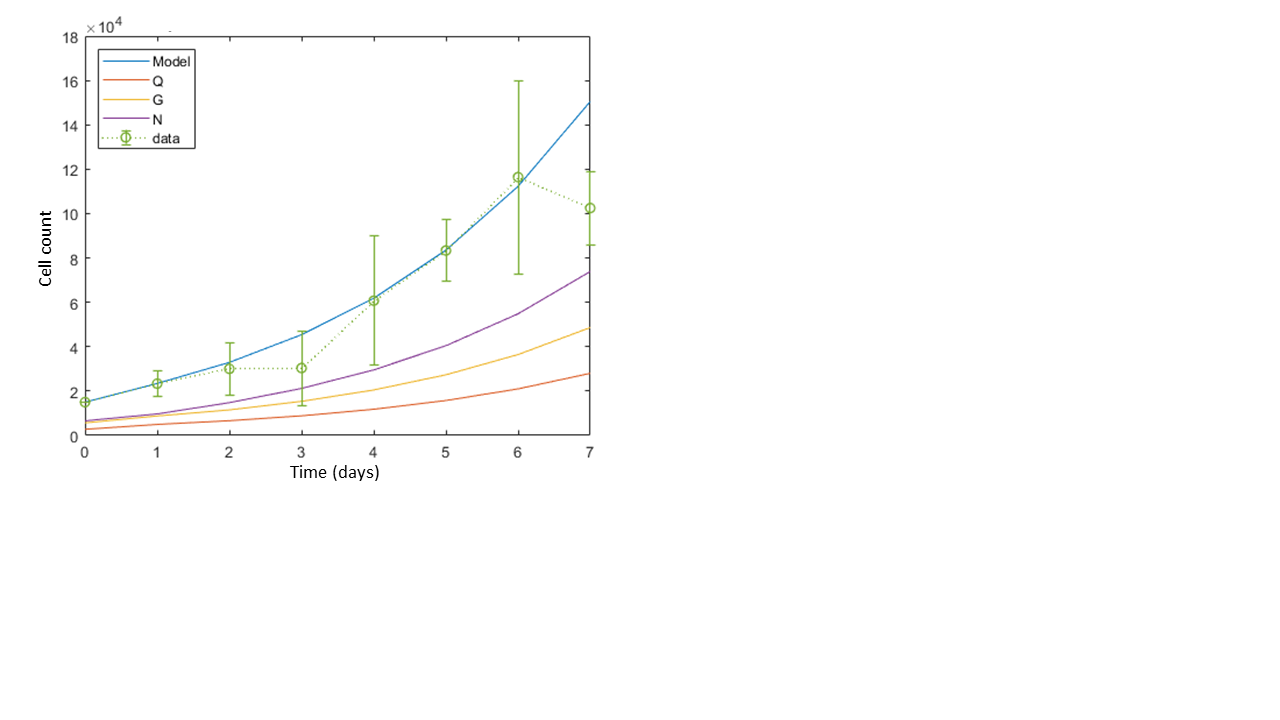


**Figure S1: Fitting result for the tumour growth parameters.** Growth curves for the sub populations of the model as well as the sum of $Q+G_{1}+N$ in blue. This fitting established the $a_{1}$, $a_{2}$ and $d_{2}$ parameters from the table above. The mean and standard error bars are representative of three experiment replicates.


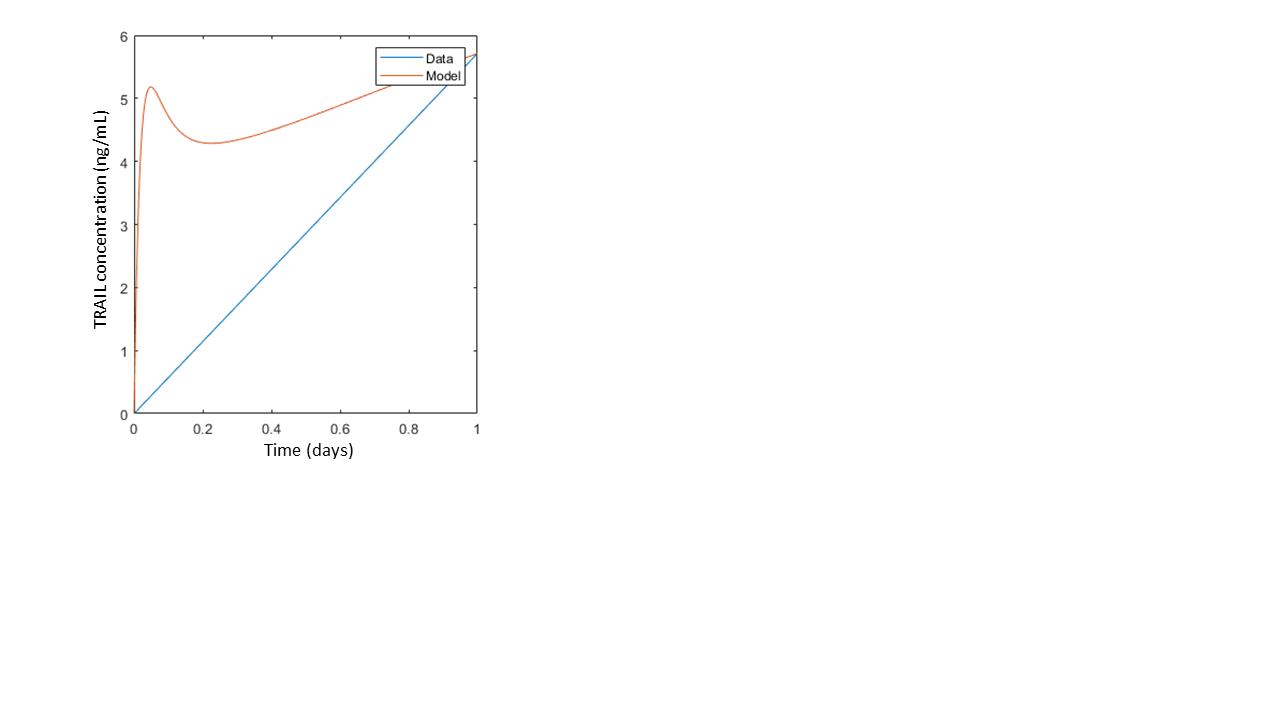


**Figure S2: Fitting result for the TRAIL production from the OV.** The data consist of 2 points only: an initial value at time t=0 and another at t=1. This fitting results in the value for $\alpha_{T}$ in the table above using data from Oh et al. ^4^.

**
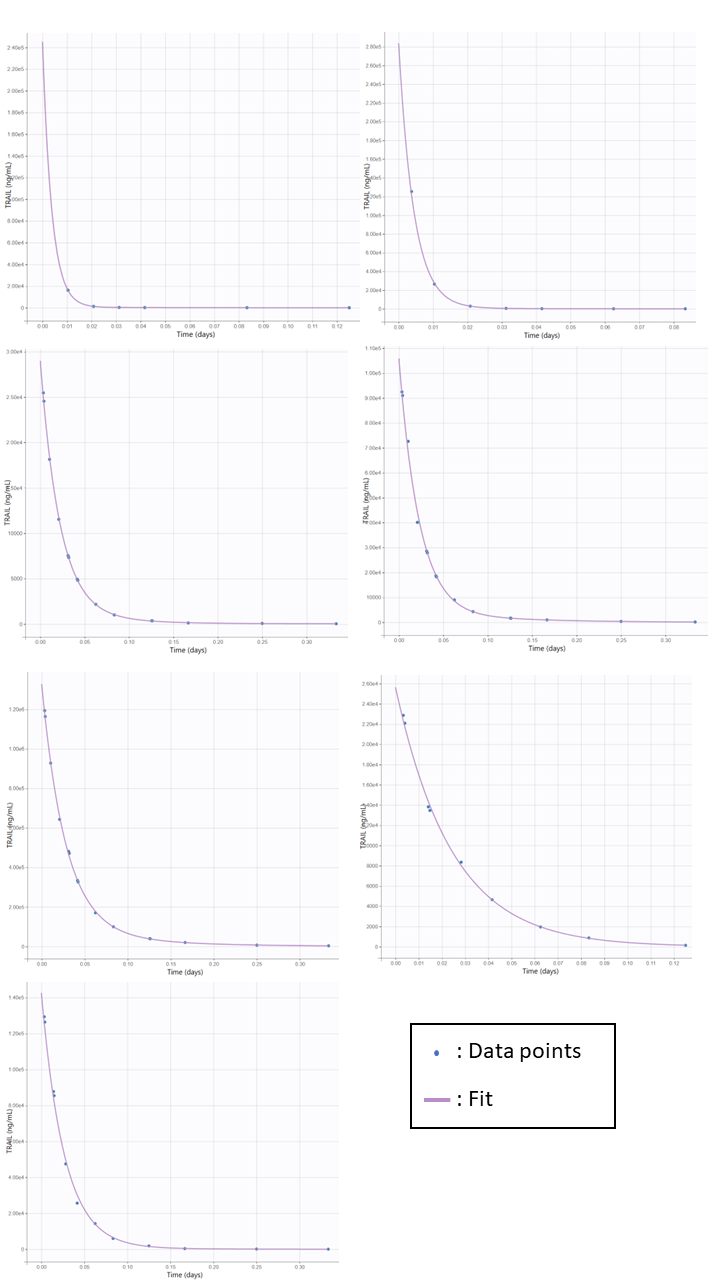
**

**Figure S3: Fitting results for TRAIL PK parameters.** Data from Kelley et al. ^5^. Seven animals of different species fitted using Monolix’s NLME model for TRAIL’s PK parameters.

**
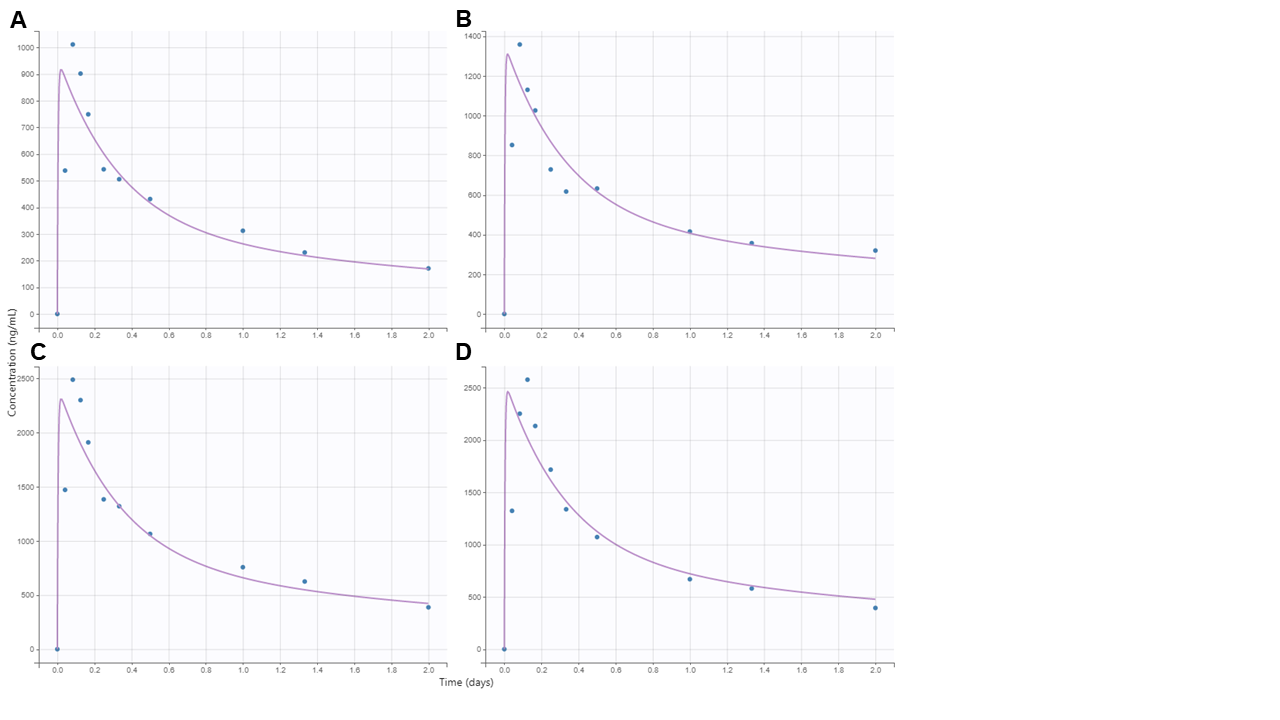
**

**Figure S4: Fitting result for oral PAC-1 PK parameters.** Data from Danciu et al. ^3^ PAC-1 concentration in two-compartment model with single oral dose of A) 250 mg, B) 450 mg, C) 625 mg and D) 750 mg.


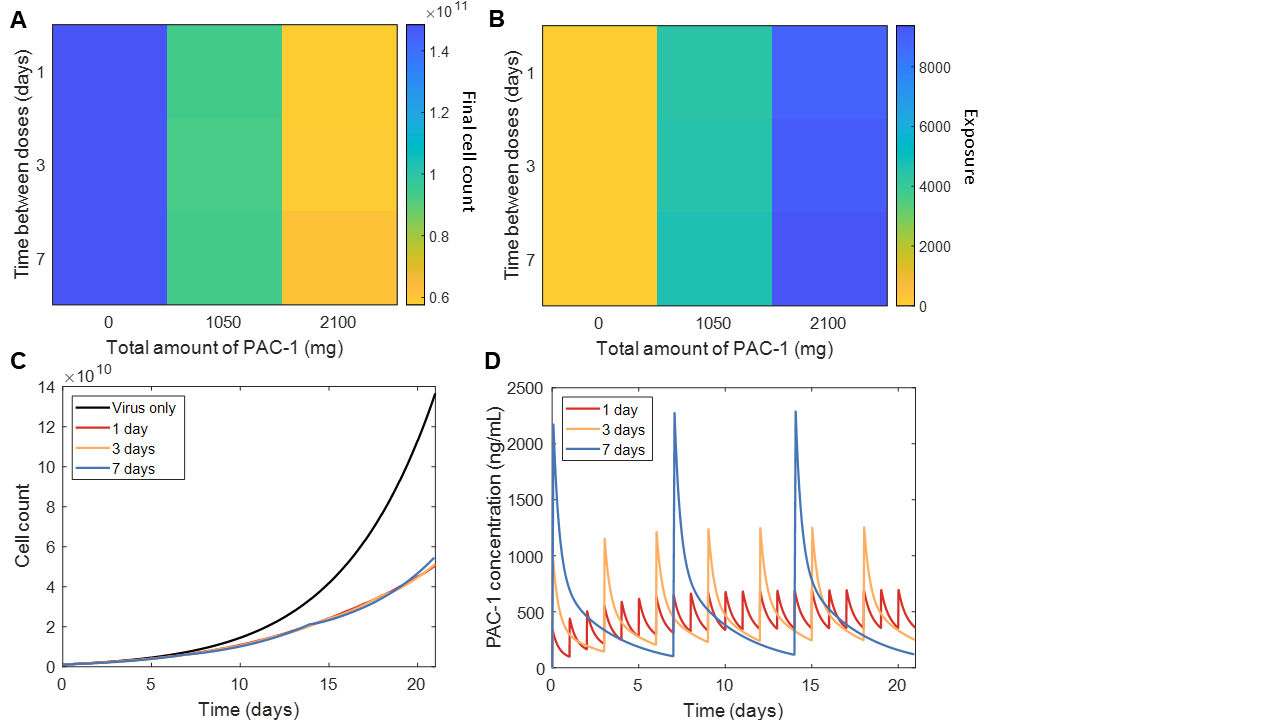


**Figure S5: Combination therapy with same total amount of PAC-1.** A) Tumour cell count after 21 days of combination therapy for total PAC-1 amount of 0 mg, 1050, mg or 2100 mg distributed over daily doses, doses every 3 days or weekly doses. B) Exposure to PAC-1 for schedules in A. C) Tumour cell count for the virus only experiment and the combination therapies using a total amount of 2100 mg of PAC-1. D) PAC-1 concentrations for 2100 mg of total PAC-1.
